# The Surgical and Serum Treatment of Puerperal Sepsis

**Published:** 1907-03

**Authors:** Lewis S. McMurtry

**Affiliations:** Louisville, Ky.


### SPECIAL ARTICLE—SELECTED.

### The Surgical and Serum Treatment of Puerperal Sepsis.

By LEWIS S. McMURTRY. A. M., M. D., Louisville, Ky.

(*From the British Medical Journal of November 3, 1906.*)

IN 1880, Pasteur, assisted by Doleris, demonstrated by cultures from cases of puerperal sepsis that streptococci were the usual infectious agents in this disease, and, by further researches, that staphylococci, and, in some cases, certain bacilli were also agents of infection. The discoveries of Lister and the development of bacteriology soon confirmed beyond question Pasteur's researches, clarified the misty atmosphere of theory and hypothesis which had so long enveloped the pathology of "puerperal fever," and established the fact, announced in 1861 by Semmelweis, that puerperal infection is identical with other wound infection. The practical results which followed the application of the Listerian surgical technique in obstetrical practice, especially in lying-in hospitals (hitherto the hotbeds of puerperal infection), were so positive and convincing that the most sceptical were persuaded, and these marvelous discoveries received acceptance generally. Every student of this subject is familiar with the names of Bumm, Winckel, Recklinghausen, Bar, Waldeyer, Kroenig, Doederlein, and others who have labored so faithfully and effectively in establishing upon a scientific basis the modern system of obstetric practice.

### PATHOLOGY.

Puerperal infection and wound infection generally are identical in origin and character, the former modified only by the anatomical relations of the wounded area, the parturient tract. Puerperal infection is contact-infection, the germs, with rare exceptions, being brought to the wounded area from without.

The organisms most commonly concerned in puerperal infection are the pyogenic bacteria so familiar in wound infection in other parts of the organism—the streptococcus and the staphylococcus. While the *bacillus coli communis,* the gonococcus, and the diphtheria bacillus are sometimes agents of infection, the familiar pyogenic organisms mentioned are the active infecting agents in the great majority of cases. The streptococcus is the cause of the severe and rapidly-fatal form of puerperal infection. Mixed infection, where two or three distinct organisms are found, is not a rare condition.

In puerperal infection the organisms invade the tissues and are diffused by means of the lymphatics and bloodvessels. The

1. Read by invitation, before the Section of Gynecology of the British Medica Association at Toronto, August 28, 1906.

extent of this invasion may be circumscribed, so that the involved area may be quite limited and the process become localised. This is more common when the infecting organism is the staphylococcus. The diffusion may be accomplished very rapidly and the entire system overwhelmed by the rapidly-multiplying organisms and associated toxins without any limitation or localisation. This severe form of infection is usually caused by the streptococcus.

The invasion of the wounded generative tract by putrefactive organisms, which do not enter the blood current but poison the system with toxins evolved, is a special form of puerperal infection known as "sapremia." The character of this infection is so distinct, both as to the organisms engaged and the toxemia produced that it should not be confounded in this consideration with the other forms of puerperal infection.

While a wound of any portion of the generative tract may afford a port of entry for infective germs, the usual site of invasion is the lining membrane of the uterus. The endometrium immediately after labor presents torn and bleeding surfaces, gaping placental sinuses newly thrombosed, and extensive vascular areas for absorption. Indeed, nature offers here an ideal culture-bed for the propagation of germ life.

In this form of endometritis the area of infection may be limited, or it may involve the entire mucous surface of the uterus. When the infection is caused by putrefactive germs, the endometrium is covered with necrotic material from which comes a foul and offensive discharge, often containing gas. An extensive invasion of the endometrium by pyogenic organisms often begets necrosis by the very intensity of infection, producing a sloughing and offensive discharge. In cases of severe infection by the streptococcus, also by the staphylococcus, there is usually no offensive odor, and the surface of the endometrium may be quite smooth, with no extensive lesions of entry. Indeed, the invasion of the endometrium by the most intense organisms, producing the most severe type of systemic infection, begets local lesions that are slight, while the lesions accompanying the ingress of putrefacive organisms, and pus-organisms of diminished virulence, are more extensive, and thereby attest the localisation of germ activity. The lymphatics so liberally distributed throughout the uterus afford the avenues of rapid extension from the endometrium. It is most pertinent in this connection to review the clinical and pathological studies of Bumm and Doederlein, showing the histological variation between cases of ordinary septic infection and cases of virulent infection. In cases wherein the pyogenic organisms are of minor degree of virulence the uterine mucosa is covered with necrotic material and large numbers of micro-organisms are embedded deeply in the membrane. Beneath is found a dense layer of cell infiltration, forming a barrier which excludes the septic organisms from further invasion. The importance of preserving this wall of defense will be referred to later in connection with certain methods of surgical treatment.

The researches of Bumm also demonstrated that in cases of septic infection wherein the mocro-organisms are most virulent, the wall of cell infiltration just described is altogether absent or very imperfectly developed. In such cases the evidences of endometritis and metritis will be slight, the uterine mucosa will be found smooth and clean, and the process of invasion is so easy and rapid that tissue resistance, either in the mucosa or beneath it, has not had time for development.

As already stated, the extension of infection from the endometrium is through the lymphatics, though the veins are also routes of invasion. Pyemia usually begins with infection of thrombi at the placental site, followed by inflammatory changes in the uterine and pelvic veins. The infecting organisms rapidly make their way from the uterine mucosa to the serous surface of the uterus, giving rise to peritonitis. The route is by means of the vessels, especially the lymphatics, and seldom by continuity of surface through the Fallopian tubes. Having reached the peritoneum, diffusion along lymph channels is boundless. The blood becomes poisoned and the entire system may be overwhelmed.

### SURGICAL TREATMENT.

With this outline of the pathological process underlying puerperal sepsis before us, we can now intelligently consider the surgical treatment of this disease. From what has preceded, it is apparent that such treatment to be effective must be applied while the infection is limited to the uterus, and, for safe procedure, to the uterine mucosa (endometritis). Hence the question of accurate diagnosis becomes a question of most vital and essential importance.

Just as soon as possible after the initial chill and rising temperature the uterus should be carefully explored, observing all necessary antiseptic precautions. Whitridge Williams recommends that sufficient of the lochia be removed for bacteriological examination, a suggestion which will prove valuable when it is practicable to do so. With the index finger the uterus should be carefully explored, and a bimanual examination will determine the mobility of the uterus and the condition of the appendages. If the examining finger discloses a smooth endometrium, free from disintegrated tissue, it will be appropriate to give an intrauterine douche of warm boiled water or sterilised salt solution. This douche should be administered by the surgeon, and with careful observance of the aseptic surgical technique. If the cavity of the uterus be filled with debris, already detected by the examining finger, an effort should be made to loosen and remove the same with the finger, afterwards applying the intrauterine douche of warm saline solution. If this procedure does not suffice to clear away the debris from the infected endometrium, we must consider a resort to the curette.

The routine use of the curette in puerperal sepsis, which is advocated by many surgeons, is both irrational and harmful. The pathological condition shows that in the most virulent type of puerperal infection the endometrium presents nothing to be removed by the curette, while its application will, by breaking through the limiting wall of leukocytes, and diffusing infective germs throughout the invaded tissues, prove most disastrous. The curette should be employed only in those cases where debris is found in the uterus, which can not be satisfactorily removed by the finger and saline douche. Its application should be followed by an intrauterine douche of warm saline solution. The inefficiency of the bichloride of mercury and carbolic acid solution has been so amply demonstrated clinically, to say nothing of their well known toxic effects, that they should be discarded entirely from intrauterine treatment. The experiments of Bumm have shown that the infecting organisms penetrate the tissues with great rapidity, and in the most virulent type of puerperal infection they have entered the uterine structures beyond the reach of bichloride injections or the curette when the initial chill marks the advancing infection. I can recall numerous cases I have seen both in hospital and private practice in which the infection advanced with increased intensity immediately after the use of the curette; whereas the curative results of curettage and douching in cases of sapremia and localised infection are most prompt and gratifying.

The operative treatment of puerperal sepsis has assumed a conspicuous place in obstetrical and gynecological literature of recent years. The existence of infected Fallopian tubes, as a complication of labor or as a sequel of puerperal infection, and their treatment by radical surgical intervention, have long been recognised by gynecologists and need not detain us at this time. The surgical treatment of parametric abscesses and inflammatory exudate, which occur usually late in the puerperium, is likewise established upon a practical basis generally known and accepted. But it is quite different as to the question of hysterectomy as a curative procedure in the early stage of infection. This subject affords a field for active discussion and much diversity of opinion. It would appear most rational that in cases of virulent infection, especially by the streptococcus, marked as it is by severe mortality, the infected uterus should be removed while the infection is limited to that organ.

That hysterectomy has a place in the treatment of puerperal infection is attested by numerous authentic cases. It is the special aim of this paper to show that the scope of this operation is restricted, and to point out the limitations which necessarily prevent its wide application. Some twelve years ago I reported a case of putrid endometritis (puerperal) in which the disease involved the deep structures of the uterus despite the treatment observed, in which I resorted to hysterectomy with prompt cure of the patient. Since that time I have seen two cases of puerperal infection in which the patients were very ill, and the process was limited to the uterus In both cases hysterectomy was done and followed by recovery. In both cases the uterine wall was the site of multiple abscesses. During this period I have seen many cases, but the three mentioned are the only cases in which I have felt justified in resorting to hysterectomy. I think my experience may be taken as that of the average surgeon engaged in special work, and may be regarded as a fair index of the restricted field for hysterectomy in the treatment of puerperal sepsis. It will be noted that in these cases operation was not undertaken in the initial stage of the disease to prevent extension of infection, but at a later stage and on account of structural changes in the uterus.

As already stated, in the virulent type of this disease, when the initial chill and rise of temperature occur, the infection has usually extended beyond the uterus. Our means of diagnosis are not so perfected that it is possible to determine the character and intensity of the infection sufficiently early to decide the important question of hysterectomy. In other words, if we resort to hysterectomy sufficiently early to arrest the disease, we subject many patients unnecessarily to a major operation; and if we defer operative intervention until the virulence of the disease is manifest, the operation will avail nothing. It is this inability to determine in the very inception of the disease (at which time alone hysterectomy will prevent extension) the character of the infection and the resistance of the invaded tissues which prevents resort to this operation. From a careful study of this subject it is apparent that, with our present means of diagnosis and prognosis, hysterectomy has no place in the initial stage of puerperal infection with a view to removing the focus of infection; and that its only proper scope is in the later stages, when the uterus has undergone pathological changes in consequence of being the storm centre, as it were, of the infecting process.

Another operative procedure which has been recommended and practised in cases of severe puerperal sepsis consists of vaginal incision and the application of antiseptic agents, preferably iodoform, to the peritoneal surfaces adjacent to the uterus. From our knowledge as to the rapid extension of the infecting process, and the superficial effect and toxic properties of the antiseptic agents used, this treatment does not appeal to favorable consideration. Its practical application has not won for it a place in the treatment of this disease.

In 1895, Marmorek introduced into the treatment of puerperal sepsis his antistreptococcic serum. By growing streptococci in human blood serum and agar, and increasing its virulence by repeated inoculation in animals, he obtained a culture of great virulence, which, by injecting into immune animals, enabled him to produce a serum claimed to be both preventive and curative. Since that time, antistreptococcic serum has been applied to the treatment of puerperal sepsis very generally throughout the world. In 1899, a committee of the American Gynecological Society made an exhaustive study of the subject, including all the cases reported up to that time. This committee made an elaborate report with the conclusion that, while the antistreptococcic serum was practically harmless, there was no evidence that it exerted a curative effect upon puerperal infection. While I have not used the serum in the treatment of this disease, I have seen it applied in numerous cases by my colleagues, and in every instance without perceptible benefit.

In order that I might present a fair estimate of the way in which this method of treatment is regarded at this time, I have requested a statement from three representative gynecologists whose clinical studies especially fit them for such consideration.

Dr. J. Whitridge Williams, of Baltimore, who was Chairman of the American Gynecological Society's Committee for the investigation of this subject, in a personal communication under date of July 20, 1906, writes:

"In the last few years renewed attention has been directed to the subject, and numerous articles have appeared from Aronsohn, Tavel, Menser, and others, which apparently show that the serum may be of value prophylactically, or in the early stages of infection, but that it is practically useless when the process has become well established. Moreover, it does not act as an antitoxin like the antidiphtheritic serum, but exerts its effect merely by stimulating the leukocytes to take up bacteria, thus increasing the opsonic power of the blood. For this reason it does not seem to me that it possesses any practical value, especially when applied after the infection has become well developed."

Dr. J. Clarence Webster, of Chicago, under date of July 21, 1906, says:

"Accurate studies of many cases by skilled observers make it evident that no better results have attended the use of the serum that have followed other methods of treatment. I have entirely abandoned its use."

Dr. Barton C. Hirst, of Philadelphia, writing on July 25, 1906, says:

"During the past year I have employed the serum in the treatment of fourteen cases of severe streptococcic infection demonstrated by blood cultures. In three cases the success was satisfactory, but in the remainder of the series the result was uncertain or entirely unsuccessful."

CONCLUSIONS.

From a careful study of the subject under consideration, it is apparent that the best results follow the simplest treatment, avoiding radical surgical intervention and facilitating drainage and elimination.

That cleansing the uterine cavity by irrigation should be given preference over curettage whenever practicable.

That on account of our inability to accurately measure the character of infection and the extent of tissue invasion in the early stage of puerperal sepsis, hysterectomy as an abortive measure of treatment is impracticable.

That antistreptococcic serum is without value in the treatment of this disease.

That puerperal infection being identical with ordinary wound infection should be considered from the standpoint of prophylaxis, and as sepsis has been eliminated from modern operative surgery, so should puerperal sepsis be reduced to the surgical standard by the application of refined surgical technique.

---

THE PRESERVATION OF HEARING.—W. Sohier Bryant states that the changes which affect the hearing most insidiously are of two kinds: those chiefly inflammatory and those chiefly due to defective ventilation; in either case the end results are much the same. Decrease of sound perception is the most delicate measure of general nervous exhaustion. The writer believes that early observation will detect insidious conditions which cause over 95 per cent. of deafness, and that judicious treatment will cure these conditions before serious impairment of hearing has taken place. He suggests that the otologist should be consulted once a year, after every cold, and whenever anything unfavorable is noticed in the ear.—*Medical Record,* March 2, 1907.

